# 2-Phenyl-2-(pyridin-2-yl)hexahydro­pyrimidine

**DOI:** 10.1107/S1600536810045976

**Published:** 2010-11-13

**Authors:** Naleen B. Jayaratna, Richard E. Norman

**Affiliations:** a Department of Chemistry, Box 2117, Sam Houston State University, Huntsville, TX 77341, USA

## Abstract

The title compound, C_15_H_17_N_3_, was prepared by reaction of benzoyl­pyridine and hexahydropyrimidine. The 1,3-diazinane ring adopts a chair conformation with one N—H group axial and the other equatorial. The axial N—H group participates in very weak hydrogen bonding to the lone pair of electrons of the N atom with the equatorial H atom producing a very weakly hydrogen-bonded dimer. The pyridine N atom accepts an inter­nal hydrogen bond from the equatorial H atom. The phenyl ring adopts an equatorial position while the pyridine ring is axial. The phenyl ring exhibits a slight twist (*ca *25°) relative to the hexahydropyrimidine ring. The pyridine ring stacks with symmetry-related pyridine rings.

## Related literature

For recent reports of the structures of other hexahydropyrimidines, see: Al-Resayes (2009 ▶); Song *et al.* (2010 ▶) and references therein. For general structural parameters for organic mol­ecules, see: Allen *et al.* (1987 ▶). For the extinction correction, see: Zachariasen (1968 ▶).
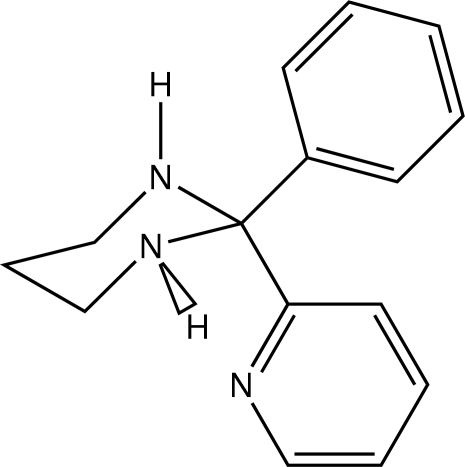


## Experimental

### 

#### Crystal data

C_15_H_17_N_3_
*M* *_r_* = 239.32
Triclinic, 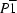

*a* = 8.2173 (2) Å
*b* = 9.0211 (2) Å
*c* = 9.1481 (3) Å
α = 97.277 (1)°
β = 94.216 (1)°
γ = 112.036 (1)°
*V* = 618.11 (3) Å^3^
*Z* = 2
Cu *K*α radiation
μ = 0.61 mm^−1^
*T* = 90 K
0.31 × 0.24 × 0.22 mm

#### Data collection

Bruker Kappa APEXII CCD area-detector diffractometer
Absorption correction: multi-scan (*SADABS*; Sheldrick, 2004 ▶) *T*_min_ = 0.823, *T*_max_ = 0.897
6458 measured reflections
2159 independent reflections
1992 reflections with *I* > 3σ(*I*)
*R*_int_ = 0.022

#### Refinement

*R*[*F* ^2^ > 2σ(*F* ^2^)] = 0.049
*wR*(*F* ^2^) = 0.166
*S* = 1.03
2159 reflections
169 parameters
H atoms treated by a mixture of independent and constrained refinement
Δρ_max_ = 0.27 e Å^−3^
Δρ_min_ = −0.18 e Å^−3^

### 

Data collection: *APEX2* (Bruker, 2006 ▶); cell refinement: *SAINT* (Bruker, 2006 ▶); data reduction: *SAINT*; program(s) used to solve structure: *SIR92* (Altomare *et al.*, 1993 ▶); program(s) used to refine structure: *TEXSAN for Windows* (Molecular Structure Corporation, 1999 ▶); molecular graphics: *ORTEPII* (Johnson, 1976 ▶); software used to prepare material for publication: *TEXSAN for Windows*.

## Supplementary Material

Crystal structure: contains datablocks global, I. DOI: 10.1107/S1600536810045976/fl2327sup1.cif

Structure factors: contains datablocks I. DOI: 10.1107/S1600536810045976/fl2327Isup2.hkl

Additional supplementary materials:  crystallographic information; 3D view; checkCIF report

## Figures and Tables

**Table 1:** Hydrogen-bond geometry (Å, °)

*D*—H⋯*A*	*D*—H	H⋯*A*	*D*⋯*A*	*D*—H⋯*A*
N3—H16⋯N1	0.90 (1)	2.365 (13)	2.7696 (12)	107.1 (10)
N2—H17⋯N3^i^	0.91 (1)	3.25 (1)	4.1179 (13)	160.2 (10)

Symmetry code: (i) 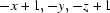.

**Fig. 1.**
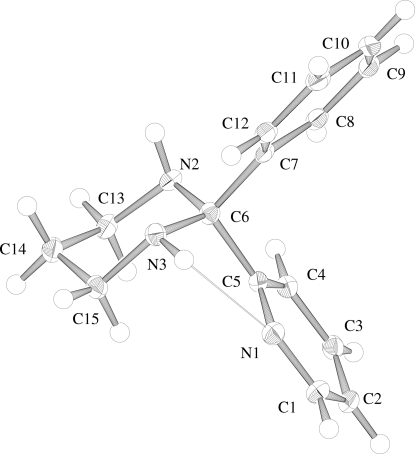
Perspective drawing of the title compound with displacement ellipsoids drawn at the 50% probability level.

**Fig. 2.**
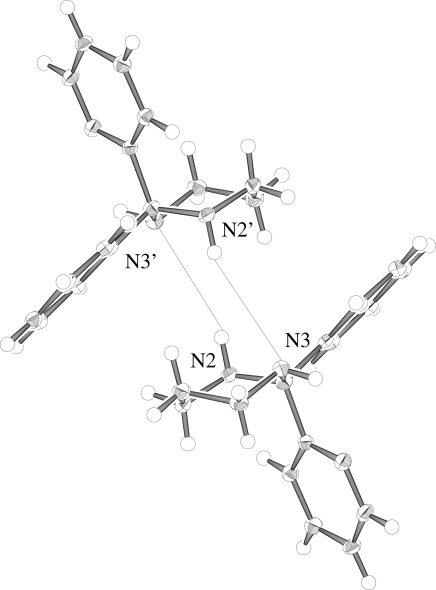
Perspective drawing of two molecules of the title compound emphasizing the weak hydrogen bonding interaction.

**Fig. 3.**
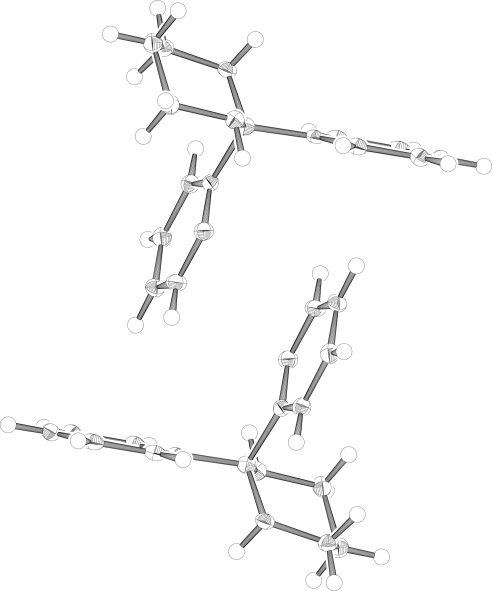
Perspective drawing of two molecules of the title Compound emphasizing pyridine ring stacking.

## supplementary crystallographic information

### Comment

During the course of an attempted preparation of a dinucleating ligand ([*N*,*N*'-bis(pyridin-2-yl)benzylidene]- propane-1,3-diamine) by reaction of two equivalents of 2-benzoylpyridine with 1,3-diaminopropane, the title compound formed instead.

The 1,3-diazinane ring adopts a chair conformation with the pyridine ring in the axial position. The pyridine nitrogen (N1) accepts an internal hydrogen bond from N3, a situation similar to that found in 2-methyl-2-(2-pyridyl)1,3-diazinane (Al-Resayes, 2009). N3 has its hydrogen atom (H16) in an equatorial position. The other amine nitrogen (N2) has its hydrogen atom (H17) in an axial position. H17 forms what appears to be a very long, very weak hydrogen bond to N3 in an adjacent molecule (1 - *x*, -*y*, 1 - *z*) (N2···N3 4.118 Å). H17 of that adjacent molecule hydrogen bonds to N3 of the original molecule, producing a very weakly hydrogen bonded dimeric pair. This is shown in Figure 2.

A more significant packing interaction is aromatic ring stacking of adjacent pyridine rings. This is shown in Figure 3 with the adjacent molecule at (-*x*, -*y*, 2 - *z*).

The phenyl ring is twisted out of the plane defined by N2, N3, C13 and C15 of the 1,3-diazinane ring by 25.1 °. This twist removes the potential steric strain between H9 of the phenyl ring and the equatorial internally hydrogen bonded H16. The phenyl ring is nearly coplanar with the C15, N3, C6 portion of the 1,3-diazinane ring (the dihedral angle is 173.9 °).

The distances within the title compound are very similar to those found in 2-methyl-2-(2-pyridyl)1,3-diazinane (Al-Resayes, 2009). Interestingly, in both compounds, the C4—C5 distance is the longest within the pyridyl ring, and in the title complex, the C7—C8 distance is the longest within the phenyl ring. Both of these bonds are relatively near N2, and are longer than the typical range (Allen *et al.*, 1987).

### Experimental

2-Benzoylpyridine (3.66 g, 20.0 mmol) was dissolved in 15 ml anhydrous ethanol. 1,3-Diaminopropane (0.835 ml, 10.0 mmol) was added and the resultant mixture was refluxed for about six and a half hours. The light brown solution was concentrated on a rotary evaporator to a light brown syrup. The syrup was allowed to stand for four weeks affording a relatively large mass of colorless crystals intermixed with a brownish semisolid material. This mixture was washed repeatedly with anhydrous ethanol at 0 ° C and decanted to remove the brownish material. The washed crystalline mass was dissolved in warm (35 ° C) anhydrous ethanol and refrigerated. After three days, colorless, cuboidal crystals were observed and proved suitable for X-ray data collection.

### Refinement

The two N—H hydrogen atoms were located in difference maps. Their positions were refined giving N—H distances around 0.90 Å. All of the remaining hydrogen atoms were placed in calculated Positions (E—H of 0.95 Å) and were refined using a riding model. All hydrogen atoms were assigned thermal parameters 1.2 times larger than the corresponding *U*_eq_ of the covalently bonded atoms.

### Crystal data

**Table:**

C_15_H_17_N_3_	*Z* = 2
*M**_r_* = 239.32	*F*(000) = 256.00
Triclinic, *P*1	*D*_x_ = 1.286 Mg m^−^^3^
Hall symbol: -P 1	Cu *K*α radiation, λ = 1.5418 Å
*a* = 8.2173 (2) Å	Cell parameters from 5072 reflections
*b* = 9.0211 (2) Å	θ = 4.9–68.4°
*c* = 9.1481 (3) Å	µ = 0.61 mm^−^^1^
α = 97.277 (1)°	*T* = 90 K
β = 94.216 (1)°	Fragment, colorless
γ = 112.036 (1)°	0.31 × 0.24 × 0.22 mm
*V* = 618.11 (3) Å^3^	

### Data collection

**Table:**

Bruker Kappa APEXII CCD area-detector diffractometer	2159 independent reflections
Radiation source: fine-focus sealed tube	2159 reflections with *I* > 10σ(*I*)
graphite	*R*_int_ = 0.022
ω and φ scans	θ_max_ = 68.4°, θ_min_ = 4.9°
Absorption correction: multi-scan (*SADABS*; Sheldrick, 2004)	*h* = −9→9
*T*_min_ = 0.823, *T*_max_ = 0.897	*k* = −10→10
6458 measured reflections	*l* = −10→10

### Refinement

**Table:**

Refinement on *F*^2^	0 constraints
Least-squares matrix: full	H atoms treated by a mixture of independent and constrained refinement
*R*[*F*^2^ > 2σ(*F*^2^)] = 0.049	Weighting scheme based on measured s.u.'s *w* = 1/[σ^2^(*F*_o_) + 0.00563|*F*_o_|^2^]
*wR*(*F*^2^) = 0.166	(Δ/σ)_max_ = 0.010
*S* = 1.02	Δρ_max_ = 0.27 e Å^−^^3^
2159 reflections	Δρ_min_ = −0.18 e Å^−^^3^
169 parameters	Extinction correction: Zachariasen (1968), equ(3) Acta Cryst.(1968) A24, p. 213
0 restraints	Extinction coefficient: 0.000012 (5)

### Special details

**Table:**

Refinement. Refinement of *F*^2^. The weighted *R*-factor *wR* and goodness of fit are based on *F*^2^, conventional *R*-factors *R* are based on *F. R*-factors based on *F*^2^ are statistically about twice as large as those based on *F*.

### Fractional atomic coordinates and isotropic or equivalent isotropic displacement parameters (Å^2^)

**Table:**

	*x*	*y*	*z*	*U*_iso_*/*U*_eq_	
N1	0.25511 (12)	0.17532 (11)	0.94163 (10)	0.0178 (2)	
N2	0.30025 (12)	0.06793 (11)	0.55486 (10)	0.0164 (3)	
N3	0.51826 (11)	0.16009 (11)	0.77508 (11)	0.0160 (2)	
C1	0.1439 (2)	0.2173 (1)	1.02090 (13)	0.0193 (3)	
C2	−0.0223 (1)	0.20332 (13)	0.96108 (13)	0.0190 (3)	
C3	−0.07658 (13)	0.14186 (13)	0.81117 (13)	0.0186 (3)	
C4	0.0366 (1)	0.09780 (13)	0.72750 (12)	0.0167 (3)	
C5	0.2016 (1)	0.11659 (12)	0.79702 (12)	0.0147 (3)	
C6	0.33262 (13)	0.06232 (13)	0.71334 (12)	0.0150 (3)	
C7	0.28971 (13)	−0.11566 (13)	0.72936 (12)	0.0145 (3)	
C8	0.1246 (1)	−0.23417 (13)	0.66514 (12)	0.0175 (3)	
C9	0.0792 (1)	−0.39534 (13)	0.68074 (12)	0.0193 (3)	
C10	0.1985 (2)	−0.44127 (13)	0.76044 (13)	0.0205 (3)	
C11	0.3625 (1)	−0.3247 (1)	0.82420 (12)	0.0197 (3)	
C12	0.4078 (1)	−0.16293 (13)	0.80897 (12)	0.0173 (3)	
C13	0.3650 (2)	0.2339 (1)	0.52058 (12)	0.0187 (3)	
C14	0.5560 (1)	0.33528 (13)	0.58810 (13)	0.0207 (3)	
C15	0.5755 (1)	0.32995 (13)	0.75312 (13)	0.0185 (3)	
H1	0.1808	0.2589	1.1238	0.023*	
H2	−0.0969	0.2351	1.0213	0.023*	
H3	−0.1898	0.1301	0.7665	0.022*	
H4	0.0025	0.0555	0.6245	0.020*	
H5	0.0428	−0.2038	0.6103	0.021*	
H6	−0.0333	−0.4744	0.6370	0.023*	
H7	0.1679	−0.5515	0.7711	0.025*	
H8	0.4444	−0.3555	0.8786	0.024*	
H9	0.5202	−0.0841	0.8533	0.021*	
H10	0.3572	0.2277	0.4157	0.023*	
H11	0.2919	0.2860	0.5582	0.023*	
H12	0.6325	0.2926	0.5417	0.025*	
H13	0.5862	0.4443	0.5733	0.025*	
H14	0.5040	0.3781	0.8004	0.022*	
H15	0.6958	0.3878	0.7947	0.022*	
H16	0.521 (2)	0.160 (2)	0.874 (2)	0.016*	
H17	0.350 (2)	0.008 (2)	0.503 (2)	0.016*	

### Atomic displacement parameters (Å^2^)

**Table:**

	*U*^11^	*U*^22^	*U*^33^	*U*^12^	*U*^13^	*U*^23^
N1	0.0186 (5)	0.0184 (5)	0.0161 (5)	0.0069 (4)	0.0032 (4)	0.0029 (4)
N2	0.0170 (5)	0.0190 (5)	0.0150 (5)	0.0088 (4)	0.0049 (4)	0.0020 (4)
N3	0.0135 (5)	0.0165 (5)	0.0171 (5)	0.0051 (4)	0.0017 (4)	0.0022 (4)
C1	0.0217 (6)	0.0206 (5)	0.0157 (6)	0.0080 (5)	0.0058 (5)	0.0021 (4)
C2	0.0181 (5)	0.0179 (5)	0.0234 (6)	0.0080 (4)	0.0099 (5)	0.0046 (5)
C3	0.0154 (5)	0.0172 (5)	0.0236 (6)	0.0058 (4)	0.0048 (5)	0.0049 (5)
C4	0.0162 (5)	0.0155 (5)	0.0173 (6)	0.0048 (4)	0.0028 (4)	0.0028 (4)
C5	0.0147 (5)	0.0129 (5)	0.0167 (6)	0.0047 (4)	0.0048 (4)	0.0036 (4)
C6	0.0118 (5)	0.0167 (6)	0.0158 (6)	0.0050 (4)	0.0017 (4)	0.0021 (4)
C7	0.0140 (5)	0.0172 (6)	0.0129 (5)	0.0064 (4)	0.0059 (4)	0.0012 (4)
C8	0.0166 (6)	0.0211 (6)	0.0144 (6)	0.0074 (5)	0.0045 (4)	0.0005 (5)
C9	0.0164 (5)	0.0206 (6)	0.0166 (6)	0.0031 (5)	0.0060 (4)	−0.0014 (5)
C10	0.0268 (6)	0.0153 (5)	0.0193 (6)	0.0073 (5)	0.0097 (5)	0.0020 (4)
C11	0.0220 (6)	0.0215 (6)	0.0192 (6)	0.0118 (5)	0.0064 (5)	0.0035 (5)
C12	0.0166 (5)	0.0191 (6)	0.0160 (6)	0.0071 (5)	0.0042 (5)	0.0010 (5)
C13	0.0189 (6)	0.0231 (6)	0.0183 (6)	0.0107 (5)	0.0071 (5)	0.0071 (5)
C14	0.0191 (6)	0.0192 (6)	0.0261 (7)	0.0081 (5)	0.0092 (5)	0.0071 (5)
C15	0.0144 (5)	0.0162 (5)	0.0254 (6)	0.0058 (4)	0.0051 (5)	0.0038 (5)

### Geometric parameters (Å, °)

**Table:**

N1—C1	1.339 (2)	C7—C8	1.401 (1)
N1—C5	1.337 (2)	C8—H5	0.95
N2—H17	0.91 (1)	C8—C9	1.388 (2)
N2—C6	1.465 (1)	C9—H6	0.95
N2—C13	1.4730 (13)	C9—C10	1.393 (2)
N3—H16	0.90 (1)	C10—H7	0.95
N3—C6	1.4702 (12)	C10—C11	1.388 (2)
N3—C15	1.4699 (13)	C11—H8	0.95
C1—H1	0.95	C11—C12	1.393 (2)
C1—C2	1.387 (2)	C12—H9	0.95
C2—H2	0.95	C13—H10	0.95
C2—C3	1.385 (2)	C13—H11	0.95
C3—H3	0.95	C13—C14	1.523 (1)
C3—C4	1.383 (2)	C14—H13	0.95
C4—H4	0.95	C14—H12	0.95
C4—C5	1.395 (2)	C14—C15	1.515 (2)
C5—C6	1.551 (1)	C15—H15	0.95
C6—C7	1.538 (1)	C15—H14	0.95
C7—C12	1.392 (2)		
			
N1···C11^i^	3.373 (1)	N3···C3^iv^	3.389 (1)
N1···C2^ii^	3.498 (2)	C1···C2^ii^	3.572 (2)
N1···C12^i^	3.501 (2)	C2···C8^ii^	3.582 (2)
N2···C4^iii^	3.381 (2)	C9···C9^v^	3.484 (2)
			
C5—N1—C1	117.65 (9)	H5—C8—C7	119.6
H17—N2—C6	108.9 (8)	C9—C8—C7	120.82 (10)
H17—N2—C13	110.7 (8)	H6—C9—C8	120.0
C6—N2—C13	113.36 (8)	H6—C9—C10	120.0
H16—N3—C6	104.7 (8)	C8—C9—C10	120.06 (10)
H16—N3—C15	107.2 (8)	H7—C10—C11	120.2
C6—N3—C15	112.82 (9)	H7—C10—C9	120.2
H1—C1—N1	118.2	C11—C10—C9	119.5 (1)
H1—C1—C2	118.2	H8—C11—C10	119.8
N1—C1—C2	123.62 (10)	H8—C11—C12	119.8
H2—C2—C3	120.9	C10—C11—C12	120.40 (10)
H2—C2—C1	120.9	H9—C12—C7	119.7
C3—C2—C1	118.23 (10)	H9—C12—C11	119.7
H3—C3—C4	120.5	C7—C12—C11	120.60 (10)
H3—C3—C2	120.5	H10—C13—H11	109.5
C4—C3—C2	118.97 (10)	H10—C13—N2	108.6
H4—C4—C3	120.6	H10—C13—C14	108.6
H4—C4—C5	120.6	H11—C13—N2	108.6
C3—C4—C5	118.85 (10)	H11—C13—C14	108.6
N1—C5—C4	122.68 (10)	N2—C13—C14	113.03 (9)
N1—C5—C6	115.06 (9)	H13—C14—H12	109.5
C4—C5—C6	122.19 (9)	H13—C14—C15	109.5
N2—C6—N3	111.48 (9)	H13—C14—C13	109.5
N2—C6—C7	107.67 (8)	H12—C14—C15	109.5
N2—C6—C5	109.10 (8)	H12—C14—C13	109.5
N3—C6—C7	109.07 (9)	C15—C14—C13	109.24 (9)
N3—C6—C5	112.26 (8)	H15—C15—H14	109.5
C7—C6—C5	107.06 (8)	H15—C15—N3	109.6
C12—C7—C8	118.61 (10)	H15—C15—C14	109.6
C12—C7—C6	122.22 (10)	H14—C15—N3	109.6
C8—C7—C6	119.15 (10)	H14—C15—C14	109.6
H5—C8—C9	119.6	N3—C15—C14	109.21 (9)
			
N1—C1—C2—C3	0.3 (2)	C2—C3—C4—C5	0.1 (2)
N1—C5—C4—C3	0.1 (2)	C3—C4—C5—C6	176.89 (9)
N1—C5—C6—N2	−155.92 (9)	C4—C5—C6—C7	−89.2 (1)
N1—C5—C6—N3	−31.8 (1)	C5—C6—N2—C13	74.1 (1)
N1—C5—C6—C7	87.8 (1)	C5—C6—N3—C15	−66.4 (1)
N2—C6—N3—C15	56.4 (1)	C5—C6—C7—C8	64.4 (1)
N2—C6—C5—C4	27.1 (1)	C5—C6—C7—C12	−114.0 (1)
N2—C6—C7—C8	−52.8 (1)	C6—N2—C13—C14	50.1 (1)
N2—C6—C7—C12	128.9 (1)	C6—N3—C15—C14	−60.0 (1)
N2—C13—C14—C15	−52.8 (1)	C6—C7—C8—C9	−178.20 (8)
N3—C6—N2—C13	−50.5 (1)	C6—C7—C12—C11	178.39 (9)
N3—C6—C5—C4	151.2 (1)	C7—C6—N2—C13	−170.05 (8)
N3—C6—C7—C8	−173.95 (9)	C7—C6—N3—C15	175.14 (8)
N3—C6—C7—C12	7.7 (1)	C7—C8—C9—C10	−0.3 (2)
N3—C15—C14—C13	56.7 (1)	C7—C12—C11—C10	−0.2 (2)
C1—N1—C5—C4	−0.1 (2)	C8—C7—C12—C11	0.1 (2)
C1—N1—C5—C6	−177.08 (9)	C8—C9—C10—C11	0.2 (2)
C1—C2—C3—C4	−0.3 (2)	C9—C8—C7—C12	0.2 (2)
C2—C1—N1—C5	−0.1 (2)	C9—C10—C11—C12	0.1 (2)

Symmetry codes: (i) −*x*+1, −*y*, −*z*+2; (ii) −*x*, −*y*, −*z*+2; (iii) −*x*, −*y*, −*z*+1; (iv) *x*+1, *y*, *z*; (v) −*x*, −*y*−1, −*z*+1.

### Hydrogen-bond geometry (Å, °)

**Table:**

*D*—H···*A*	*D*—H	H···*A*	*D*···*A*	*D*—H···*A*
N3—H16···N1	0.90 (1)	2.365 (13)	2.7696 (12)	107.1 (10)
N2—H17···N3^vi^	0.91 (1)	3.25 (1)	4.1179 (13)	160.2 (10)

Symmetry codes: (vi) −*x*+1, −*y*, −*z*+1.
